# Advances in Optical Aptasensors for Early Detection and Diagnosis of Various Cancer Types

**DOI:** 10.3389/fonc.2021.632165

**Published:** 2021-02-25

**Authors:** Qurat ul ain Zahra, Qaiser Ali Khan, Zhaofeng Luo

**Affiliations:** ^1^ Core Facility Center for Life Sciences, Department of Biochemistry and Molecular Biology, School of Life Sciences, University of Sciences and Technology of China, Hefei, China; ^2^ Hefei National Lab for Physical Sciences at the Microscale and the Centers for Biomedical Engineering, University of Science and Technology of China, Hefei, China; ^3^ Institute of Chemistry of New Materials, Universität Osnabrück, Osnabrück, Germany

**Keywords:** optical aptasensors, early cancer diagnostics, cancer biomarkers, fluorescence aptasensors, chemiluminescence, SERS cancer aptasensors, colorimetric aptasensors, SPR aptasensors

## Abstract

Cancer is a life-threatening concern worldwide. Sensitive and early-stage diagnostics of different cancer types can make it possible for patients to get through the best available treatment options to combat this menace. Among several new detection methods, aptamer-based biosensors (aptasensors) have recently shown promising results in terms of sensitivity, identification, or detection of either cancerous cells or the associated biomarkers. In this mini-review, we have summarized the most recent (2016–2020) developments in different approaches belonging to optical aptasensor technologies being widely employed for their simple operation, sensitivity, and early cancer diagnostics. Finally, we shed some light on limitations, advantages, and current challenges of aptasensors in clinical diagnostics, and we elaborated on some future perspectives.

## Background and Introduction

Cancer is one of the main life-threatening concerns both in developed and developing countries around the world. Stomach, breast, liver, colorectal, and lung cancer are the most common cancer types causing a high mortality rate every year (1). Abnormal, uncontrolled cell division, apoptotic resistance, and accumulation of increasing genetic mutations are the leading causes of tumor development. Specific proteins are expressed on the surface of cancerous cells, which are not expressed by healthy normal cells or sometimes expressed in smaller amounts. These surface proteins are known as cancer biomarkers and are used to detect cancer (2). Since cancer is a deadly disease, sensitive and early-stage diagnostics can make it possible for the patients to get through the best available treatment options to combat this menace for longer survival (3). Currently, different cancer diagnostic tests are available including mammography, colonoscopy, cervical cytology, prostate-specific antigen, immunohistochemistry, molecular detection, cancer imaging (IHC), and many more, and all have some associated limitations that may produce unauthentic results (4, 5). Bing et al. screened a novel BG2 (G-rich) aptamer by systematic evolution of ligands by exponential enrichment (cell-SELEX). Their ssDNA aptamer as molecular probe could isolate alkaline phosphatase heterodimers (from cell lysate) present on the surface of various cancer cells (both *in vivo* and *in vitro*) (6). In another report, Bing et al. introduced a wy-5a aptamer that specifically binds prion proteins (reference markers) on tumor cells and tissues. They demonstrated that it could serve well as a probe in diagnostics and therapy of breast and prostate cancers (7). Consequently, there was an increasing demand to develop a precise, cost-effective, sensitive, and early-stage cancer detection method for various cancer types to prevent malignancies.

Oligonucleotide aptamers are single-stranded, short, nucleic acid (DNA or RNA) sequences with an ability to rearrange into a three-dimensional unique structure for highly specific binding to their particular targets ranging from proteins (8, 9) to even whole cells (10). A variety of aptamers in the last two decades have been screened against different cancer biomarker proteins overexpressed on the tumor surface. Some high-affinity aptamers specific for certain cancer biomarkers are used in early cancer diagnostic sensing platforms (11). The targets are purified for further detection by aptamers, which are used as ligands making targets a potential new biomarker. The binding of aptamers to unknown molecular signatures of a particular cell leads to the discovery of potential biomarkers (12). Aptamers as biorecognition elements in biosensors have shaped a new kind of sensing technology known as aptasensors (13) and exhibit an exceptional recognition ability against their particular target types (14). They can be used in a variety of applications such as the detection of chemicals, disease markers, and foodborne pathogens (15). Aptasensors are classified based on their signal transduction modalities including electrical, micromechanical, mass-sensitive, and optical aptasensors (16). Numerous optical aptasensor types have been developed to bind and identify different cancer biomarkers or cells. In this mini-review, we have focused on recent advances in different types of optical aptasensors being used for early detection of cancer.

## Optical Aptasensors in Early Cancer Detection

Optical methods are advantageous because they show a quick response, simple operation, and high sensitivity. Optical aptasensors involve aptamers as a biorecognition element along with various optical approaches as signal transduction element (17). Optical aptasensors can be classified based on their luminescence changes and light absorption as a result of interaction with different analytes. These aptasensors usually have minute reagent requirements, cost-effectiveness, simple labeling, and swift procedures (18). Optical aptasensors are categorized based on different optical detection methods used to diagnose different cancer types at early stages and are discussed below. Aptamer name, their particular target cancer biomarkers/cells, and the limit of detection/sensitivity have been summarized in **Table 1** for all (2016–2020) reports discussed in this mini-review.

**Table 1:** Aptasensors reported for early cancer detection.

Aptamer	Target/Analyte	LOD/sensitivity	Strategy	Reference
AS1411	MCF-7	10 cells	col	(19)
AS1411, and MUC1	MCF-7	5 cells	SERS	(20)
BG2	IAP-PLAP heterodimer proteins on CTRMs	92%	col	(21)
CDs	CA125 marker and OVCAR-3 cells	5 × 10^-7^ ng/mL, 400 cells/mL	F	(22)
CD63	MCF-7 cells	5 × 10^3^ exo/mL	SPR	(23)
CD63	MCF-7 cells	13.52 × 10^8^ part/mL	col	(24)
CD63	MCF-7 cells	5.2×10^8^ part/mL	col	(25)
CD63, HER2, integrin αvβ6	CD63 cells	7.7 × 10^3^ part/mL	col	(26)
CEA	CEA biomarker	0.034ng/mL	F	(27)
CEA	CEA biomarker	0.56 pM, 18.8pM	ECL-SPR	(28)
CEA, CD631, H2 and PSMA	SKBR3, T84 and LNCaP biomarker	32×10^3^exo/mL for SKBR3, 73 exo ×10^3^/mL for T84, and 203×10^3^ exo/mL for LNCaP	SERS	(29)
CEA, PSA, Thr	CEA biomarker	——	F	(30)
**CH-1, CH-2**	CEA biomarker	0.58 ng/mL	CL	(31)
HAP	CCRF-CEM cells	25 cells	F	(32)
HApt	HER2 biomarker	0.0904 fM	F	(33)
H1, H2	Ramos cells	230 cells/mL	ECL	(34)
HL-60 cell	HL-60 cancer cells	150 cells/mL	ECL	(35)
mamA	MCF-7 cells	49 cell/mL	SPR	(36)
MUC-1	Gastric cancer exo	4.27 × 10^4^/mL	F	(37)
MUC-1	MCF-7 cells	100 cells/mL	SPR	(38)
MUC1	MUC1 biomarker	0.1 U/mL	SERS-col	(39)
P1 (EpCAM)	CTCs (on MCF-7 cells)	10 cells	col	(40)
PSA	PSA biomarker	——	PEC	(41)
PSA	PSA biomarker	0.002ng/mL	LS	(42)
PSA	PSA biomarker	0.00071 ng/mL, 0.77ng/mL	ECL-SPR	(43)
PSA	PSA biomarker	0.00017ng/mL	ECL	(44)
PSA, polyA	PSA biomarker	0.02ng/mL	col	(45)
PSA, Hemin	PSA biomarker	0.1 ng/mL	CL	(46)
PSMA, HER2	HER2 biomarker	92.31%	F	(47)
PSMA, HER2, and AFP	LNCaP, SKBR3, and HepG2 biomarkers	26×10^3^part/mL for (LNCaP), 72×10^3^ part/mL (SKBR3), 35×10^3^part/mL (HepG2)	SERS	(48)
Sgc8c	PTK-7 biomarker on Hela cells	——	SERS-F	(49)
Sgc8c	CEM cells (leukemia)	46 cells	F	(50)
Sgc8c	CCRF-CEM cells	10 cells/mL	F	(51)
TLS11a	HepG2 cells	——	F	(52)
VEGF	VEGF165 biomarker	0.01 ng/mL	col	(53)

F, Fluorescence; CL, chemiluminescence; ECL, electrochemiluminescence; SPR, surface plasmon resonance; CTCs, circulating tumor cells; SERS, surface-enhanced Raman scattering; col, colorimetric; part, particles; Exo, exosomes; CTRMs, circulating tumor-related materials; PEC, photoelectrochemical; LS, light scattering. All units in LOD/sensitivity column are converted to make it more understandable and comparable.

### Fluorescence-Based Optical Aptasensors

Fluorescence is an optical approach commonly employed to construct aptasensors for their low costs, high sensitivity, operation simplicity, and high efficiency (54). Lei et al. introduced a “nanodoctor” known as “smart split aptamer-based activatable theranostic probe (SATP)” for *in vivo* cancer imaging that not only can activate fluorescence signals as a result of interaction with its analyte but also releases the drug (50). A graphene oxide-based label-free aptasensor for quantitative diagnostics of rare CCRF-CEM cells was employed by Xiao et al. CTCESA-based (cell-triggered cyclic enzymatic signal amplification) fluorescent aptasensors show a better selectivity and sensitivity for clinical and preclinical cancer detection in comparison to normal fluorescence-based aptasensors (32). Hamd-Ghadareh et al. constructed an antibody-ssDNA aptamer-based fluorescence sandwich-type ultrasensitive biosensor for CA125 early detection (22). A fluorescent “turn on” aptasensor based on fluorophore-labeled protein-aptamers and MoS2 (molybdenum disulfide) nanosheets was assembled by Zhao et al. for a highly sensitive and rapid CEA protein detection (27). In addition, Lai et al., Tan et al., and many others have recently published articles based on fluorescent aptasensors (51, 52). Another label-free, versatile “turn on” fluorescent aptasensor for HER2 early detection was fabricated by Zhang et al. (33). Exosomes for gastric cancer detection can be efficiently identified by a method designed by Huang et al. (37). A multiplex, competitive aptasensor based on fluorescent nanoparticles count was proposed by Pei et al. to detect various cancer biomarkers (30). Li et al. designed a platform not only efficient in exosomal protein profiling but also filling the technological innovation gap to facilitate the exosomal detection assays and shed light on methods for early detection of cancer such as liquid biopsy (47). An aptamer dependent fluorescence polarization technique was established by Zhang et al. that allows direct quantification of exosomes in human plasma without separation. It minimizes the operation time by simplifying the quantification without losing exosomes from the sample during separation (55).

### Chemiluminescence Based Optical Aptasensors

A phenomenon where emitted light from a substance is not because of heat is called luminescence, which can be categorized based on the energy (trigger) sources into electrochemiluminescence (ECL) and chemiluminescence (CL). Both are widely used in the development of aptasensors employed in cancer biomarkers detection (56). Several CL aptasensors for early cancer detection have already been introduced (57). It is considered the most sensitive optical approach because of excellent results (58). Jie and Jie described a quantum dots (QDs) nanocluster based ECL signal probe with a great potential for early cancer diagnosis in clinical samples (34). Wang et al. designed a ratiometric dual-signaling electrochemiluminescence aptasensor exhibiting good reproducibility, high selectivity, and stability against their target cancer cells among non-target cancer cells (35). A simple, all in one, cost-effective, easy to operate, and portable medical platform to be used in hospitals and homes was designed by Khang et al. for early-stage breast cancer diagnostics (31). Another competitive, novel GO@AuNRs-GOD-SA nanoprobe based ECL aptasensor for PSA detection was demonstrated by Cao et al. (44). It is considered to be an excellent advancement for the detection of trace-level disease biomarkers. Kim et al. fabricated a fast biosensor, based on a dual aptamer system connected by a 5 adenine linker, to be used for rapid and accurate PSA quantification (46).

### Surface Plasmon Resonance (SPR) Based Aptasensors

Surface plasmon resonance (SPR) based biosensing is advantageous because of label-free and kinetic studies exploring properties that are not offered by many other systems, giving direct and real-time detection of targets. SPR based assays are widely used to detect several types of cancer cells and biomarkers due to their high sensitivity (59). Li et al. proposed that MUC-1 aptamer (Mucin 1 protein) functionalized gold nanorods (AuNRs) have the ability to specially recognize MCF-7 cells *via* specific interactions that can be further processed by their unique localized surface plasmon resonance (LSPR) spectra. Their biosensor can be employed to detect human breast cancer at early stages (38). Electrochemical and SPR assays were combined by Guo et al. to examine the detection kinetics, which revealed significant outcomes for CEA detection by using their developed aptasensor based on the AgNCs@Apt@UiO-66 nanocomposite. Their SPR aptasensor is considered to have good performance, regenerate ability, selectivity, acceptable reproducibility, high sensitivity, and stability (28). A bi-functional (electrochemical-SPR) aptasensor with exceptional electrochemical action of MoS2QDs@g-C3N4 nanosheets and good SPR enactment of CS-AuNPs was combined by Duan et al. to make a 2D MoS2QDs@g-C3N4@CS-AuNPs nanocomposite. The authors expected satisfactory results of their sensor for the detection of cancer markers in clinical applications (43). A highly effective and sensitive SPR aptasensor for exosomal detection was invented by Wang et al., which is based on dual AuNPs assisted signal amplification. The approach finds promising practical applications in clinical and biological studies (23). Loyez et al. devised multiple narrowband resonances (near-infrared wavelength range), an all-fiber SPR aptasensor that takes five minutes for the detection of metastatic breast cancer cells. Supplementary addition of functionalized AuNPs enhances the 2-fold performance of the aptasensor (36).

### Surface-Enhanced Raman Scattering (SERS)-Based Aptasensors

SERS spectroscopy has emerged as a promising tool for characterization in the field of nanoscience, i.e., widely investigated in cancer-related applications (60–62). The major advantages of SERS imaging are the mapping of a sample with a high spatial resolution (< 0.5 microns in the visible range) and the capability of multiplexed analysis (63). The ultrasensitive vibrational spectroscopic technique SERS can be used to detect several target molecules in a single experiment (60, 64). Ning et al. synthesized the aptamer-based SERS detection probes based on gold–silver–silver core-shell–shell nanotrepangs (GSSNTs) nanotags and magnetic beads for simultaneous detection of multiple cancer-related exosomes: the biomarkers (PSMA, Her2, and AFP proteins) for the prostate cancer cell line (LNCaP), breast cancer cell line (SKBR3), and hepatocellular cancer cell line (HepG2) (48). For the simultaneous detection of multiple kinds of exosomes (SKBR3, T84, and LNCaP), three different SERS probes types were designed to have three different types of Raman reporters and aptamers by Weng et al. and the principle of SERS detection (29). Liang et al. fabricated a series of aptamer-charged SERS probes (AS1411 and MUC1) for targeting cancer cells (MCF-7), and their results show the limit of detection (LOD) up to five cancer cells (20). Lately, the SERS spectroscopy method has been combined with other techniques for attaining maximum information from a sample. Li et al. fabricated a SERS-colorimetric dual-mode aptasensor for cancer biomarker MUC1 detection. The SERS probes were fabricated by using modified gold-silver core-shell nanoparticles with Raman reporters and the sequence of MUC1. The SERS probes report both SERS and colorimetric signals simultaneously (39). Bamrungsap et al. combined SERS and fluorescence nanotags assembled-system using a layer-by-layer process. The nanotags consisting of gold-silver nanorods, aptamers, and fluorophore-labeled aptamer for SERS signal generation, targeting ligands and fluorescence imaging, respectively. The dual-mode sensor system was successful for highly sensitive and specific cancer (cervical cancer) diagnostics (49).

### Colorimetric Aptasensors

Colorimetric-based aptasensors have been used for the detection of disease biomarkers, due to their simplicity, ease of use, accessibility, and point-of-care detection (65, 66). The colorimetric method is a promising technique due to the possibility of detection by simply visual color change (67). Xu et al. developed a highly sensitive colorimetric-based aptasensor for the detection of exosomes obtained from breast and pancreatic cancer cells. In this novel approach, the specific detection was accelerated by horseradish peroxidase (HRP) accelerated dopamine polymerization, and sensitivity was enhanced by *in situ* deposition of polydopamine around exosomes particles (26). Shayesteh et al. developed a label-free colorimetric aptasensor, using poly-adenine aptamer and gold nanoparticles for sensitive detection of prostate-specific antigen (PSA) tumor marker. The concentration of PSA (5ng/ml) was detected by the naked eye with the color change (45). Dong et al. proposed a novel highly selective calorimetric based aptasensor strategy for detection of the vascular endothelial growth factor165 (VEGF165) in human serum (53). Colorimetric aptasensor based on gold nanoparticles aggregation developed by Borghei et al. was shown to have good results for the detection of rare circulating cancer cells. In this method, aptamer desorbed from solution due to specific binding of AS1411 aptamer to cancer cells, which resulted in the solution color change from purple to red (19). Xia et al. designed a fast and label-free DNA-capped-Single-Walled Carbon Nanotubes based aptasensor for exosomes detection through visible inspection. The exosomes were obtained from MCF-7 and breast cancer patient’s serum. The ability to detect exosomes in a homogenous system in combination with excluding complicated rinsing procedure is the key advantage of this proposed method (25). Wang et al. demonstrated single-stranded DNA (ssDNA) with graphitic carbon nitride nanosheets (g-C3N4 NSs) hybrid aptasensor for the colorimetric detection of exosomes originated by a breast cancer cell line (MCF-7) and a control cell line (MCF-10A). The intrinsic peroxidase-like activity of g-C3N4 NSs was enhanced by ssDNA (24). Shen et al. fabricated a colorimetric aptasensor for the detection and isolation of circulating tumor-related materials and is based on aptamer functionalized magnetic nanoparticles and endogenous alkaline phosphatase signal amplification. Their method exhibited great potential for clinical samples and is considered to find promising applications in point-of-care testing (21).

### Other Optical Aptasensors

Terahertz radiation (THR) finds useful applications in cancer imaging (68). To overcome THR shortcomings regarding cancer cell and biomarkers detection, new technology has recently emerged known as terahertz chemical microscopy (TCM). However TCM has been reported to detect metastatic breast cancer cells, and only limited reports has been published (69). A photoelectrochemical (PEC) aptasensor fabricated by Zhou et al. (2017) is based on reduced graphene oxide-functionalized iron oxyhydroxide (FeOOH-rGO) as the photoactive material for the detection of PSA (prostate-specific antigen). Accuracy, specificity, and stability of the system were comparable to the commercially used PSA ELISA (enzyme-linked immunosorbent assay) kit (41). Liu et al. (2019) reported an ultrasensitive, activatable light-scattering (LS) method for PSA detection in real samples. The working mechanism of the aptasensor is based on target stimuli-responsive aggregation of AuNPs, which are responsible for lighting up the light-scattering signals (42).

## Aptasensors in Clinical Diagnostics

An early cancer diagnosis is particularly an active research area because early detection can help to improve patient survival and disease prognosis. For this purpose, very sensitive and stable methods are needed for early cancer diagnosis (70). The main advantages of using aptasensors for clinical diagnostics are high selectivity and specificity, and low cost of production (71). The stability, ability of easy modification, and capability of fast development (animal-free) make nucleic acid aptamers detection methods widely functional compared to traditional antibody-based detection methods. And the nucleic acid aptamers can be used against a wide spectrum of targets (71, 72). The smaller size of aptamers compared to antibodies improves transport and tissue penetration (72). However one of the main disadvantages of the aptasensor is restricting each aptasensor to one marker or cell type (73). The development of an increasing number of published articles on aptamers for oncological diseases detection shows increased interest and progress in aptamer technology. Despite all the advantages, traditional immunoassays are still the dominant technology in the field of clinical diagnostics (70, 71). Nevertheless, this knowledge utilization for clinical practices has been challenging and the process has been very slow. There are many challenges if the aptasensor based sensing platform is to be used commercially. For example, improved signal-to-noise ratio and a high level of confidence in signal detection must be recognized (74). The compatibility of aptasensor assay with current equipment of diagnostics units is also an issue and is for the reason of the fragility of aptasensors (75). Many published reports investigated the sensing in buffer or diluted biological fluids; however, the goal should be the detection of biomarkers in a raw biological fluid. The cost of the whole sensing system should also be considered, for example, the cost of TCM components (laser) is expensive and uses a bulky femtosecond laser setup (11). Aptasensors after resolving all the above-discussed obstacles can be one of the most important early cancer detection tools (74).

## Conclusion and Future Perspectives

Since early cancer detection has significant roles to increase available treatment options for the longer survival of patients, advances in various types of optical aptasensors for the detection of cancer cells and biomarkers or exosomes have been comprehensively summarized in this mini-review. Fluorescence-based label-free/labeled (e.g., FRET-based) aptasensors in combination with different nanomaterials/dyes, etc. as fluorophores, and quenchers to quench (change) the fluorescence properties as a result of specific interactions, have gained increasing attention. ECL and CL owing to their wide-ranging calibration and low background signals have recently been broadly exploited. Other types of optical aptasensors based on SPR, TCM, and SERS, etc. are also highly recommended for early-stage cancer diagnostics. Colorimetric methods combined with several different latest strategies (e.g., nanoparticles, etc.) implicate the simplest aptasensors and can be analyzed easily with the naked eye.

Several optical aptasensors reported for cancer early detection exhibit good performance in terms of selectivity and sensitivity, yet commercially available aptasensors just appear as the tips of some icebergs when we compare them to the mighty academic literature available in this area. Some new methods are still in lab trials with the early results favoring their commercial applications outside labs. However, some important technological issues and challenges still need to be addressed or improved. First, only a limited number of good specificity/sensitivity aptamers are available against a certain type of cancer cells/biomarkers, and more aptamers need to be screened that could target multiple cancer biomarkers without any complexity or off-target recognition in biological samples. Second, aptasensors need further investigations for clinical applications with real, undiluted (raw) biological samples with a primary focus on aptamer specificity, high sensitivity, cost-effectiveness, and simple operation. Overall, it is evident that the full bloom of optical aptasensor technology for cancer diagnostics is still on the way to a bright future.

## Author Contributions

All authors listed have made a substantial, direct, and intellectual contribution to the work and approved it for publication.

## Funding

We acknowledge the financial support from “the Fundamental Research Funds for the Central Universities (No. YD2070002013)” and the National Natural Science Foundation of China (No. 31570755).

## Conflict of Interest

The authors declare that the research was conducted in the absence of any commercial or financial relationships that could be construed as a potential conflict of interest.
